# Hepatitis B vaccination in Chinese infants (2002-2006, Guangdong): long-term effectiveness in adulthood and biological features of HBV infection

**DOI:** 10.3389/fimmu.2026.1787290

**Published:** 2026-04-29

**Authors:** Qiao Liao, Bochao Liu, Fenfang Liao, Ru Xu, Min Wang, Zhengang Shan, Jieting Huang, HuiShan Zhong, Yongshui Fu, Huaqin Liang, Xia Rong

**Affiliations:** 1 Institute of Blood Transfusion and Hematology, Guangzhou Blood Center, Guangzhou Medical University, Guangzhou, Guangdong, China; 2 The Key Medical Laboratory of Guangzhou, Guangzhou, Guangdong, China; 3 Department of Hematology, School of Medicine, The Second Affiliated Hospital of South China University of Technology (Guangzhou First People’s Hospital), Guangzhou, Guangdong, China

**Keywords:** anti-HBc, anti-HBs, blood donor, gene mutations, vaccination

## Abstract

**Background:**

Hepatitis B virus infection is a serious health concern in China. The National Immunization Program has implemented universal hepatitis B vaccination for infants for over two decades. The serological characteristics of hepatitis B virus and the causes of infections among adult blood donors born after 2002 remain undetermined.

**Methods:**

Qualified blood donors born between 2002 and 2006 from Guangdong were screened for antibodies against hepatitis B surface antigen (anti-HBs) and hepatitis B core antigen (anti-HBc). Donations positive for HBsAg were subjected to various tests, including HBV serological markers, quantitative PCR (qPCR), and nested PCR, to further analyze the molecular characteristics of HBV DNA. Notable mutations were identified through comparison with HBV reference sequences obtained from blood donors.

**Results:**

This study enrolled 5,487 blood donors with complete results for anti-HBs and anti-HBc testing. Overall, the prevalence of anti-HBs was 47.73% in males and 46.16% in females, while the prevalence of anti-HBc was 10.58% in males and 12.79% in females. Among blood donors born between 2002 and 2006, the prevalence of anti-HBs declined progressively with more recent birth years, decreasing from 54.55% to 40.23%, while the prevalence of anti-HBc remained relatively stable at around 11%. The prevalence (56.54%) and level of anti-HBs in the Pearl River Delta were significantly higher than those in the eastern, western, and northern regions of Guangdong (P<0.001). Quantitative analysis revealed that anti-HBs levels were significantly higher in the anti-HBc+ cohort than in the anti-HBc- group. Furthermore, whole genome sequences genotyping of 72 HBsAg+ samples revealed that 75% (54/72) were genotype B and 25% (18/72) were genotype C. Nine HBV-related point mutations were detected, including four previously reported variants and five novel substitutions. Additionally, six insertions/deletions were observed in genotype B/C. All these mutations were mapped to critical functional regions within the PreS1, Core, X, and S genes.

**Conclusion:**

Only 47.13% of young adults vaccinated at birth under the national immunization program retained protective antibodies at the time of testing, indicating this age group vulnerable to HBV infection. This decline has consequently increased the risk of breakthrough infection, necessitating booster vaccination among young adults in Guangdong.

## Introduction

HBV infection is a serious liver infection and remains a global public health issue that requires urgent response. HBV can cause both acute and chronic infections, thereby increasing the risk of patients developing liver cirrhosis and liver cancer, which are the main causes of death (1, 2). China has one of the largest HBV carrier populations in the world, with approximately 120 million carriers, including 86 million chronically infected individuals, accounting for nearly one-third of the global infected population (3). Since there is currently no clinical cure for hepatitis B, vaccination remains especially crucial. Since 2002, China has officially incorporated the hepatitis B vaccine into the National Immunization Program (NIP) (4, 5). Between 1992 and 2014, HBsAg prevalence decreased by 52%, dropping to 0.3%, 0.9%, and 4.4% in age groups 1-4, 5-14, and 15–29 years, respectively (6). Given China’s large population, continuous monitoring of HBV epidemiology is essential to guide vaccination strategies, ensure public health, and reduce disease burden.

To reduce the treatment burden of liver diseases caused by hepatitis B, both the US CDC and the Chinese Liver Association recommend that adults aged ≥18 years undergo HBV screening at least once in their lifetime. During screening, test for HBsAg, anti-HBs, and total anti-HBc. This aims to achieve early detection and treatment of infected individuals in the population (7). Although the hepatitis B vaccination NIP has been implemented, the dynamic changes in anti-HBs levels among the vaccinated population over time with respect to age, as well as the timing of additional hepatitis B vaccinations, remain unclear. In China, the national immunization program recommends a complete three-dose HBV vaccine series for all infants, with the first dose administered within 24 hours of birth. A booster dose is recommended for high-risk adults or those with an inadequate immune response (anti-HBs < 10 IU/L) (8). Despite this immunization strategy, cases of breakthrough HBV infection in vaccinated individuals have been increasingly reported. While mother-to-child transmission (MTCT) has been identified as the primary route in such cases (9–12), the contribution of other transmission routes remains unclear. Possible reasons may include a decline in vaccine protection efficacy (anti-HBs < 10IU/L), immunosuppression, and immune escape due to viral mutations. Such breakthrough infections may increase the risk of Occult Hepatitis B Virus Infection (OBI), thereby posing new challenges to blood safety (13, 14). Therefore, continuous monitoring and evaluation of the immune response after vaccination, and strengthening screening and management of blood donors, are crucial for ensuring blood safety and preventing HBV transmission. This study focuses on the cohort of individuals born after the implementation of the hepatitis B vaccination NIP. This cohort is currently attending college. College represents the final stage of formal education for most students and is also a period when sexual activity increases, significantly raising the risk of infection (15–19). Understanding the serological characteristics of hepatitis B virus in blood donors among college students in Guangdong Province and the biological characteristics of breakthrough infections will provide a scientific basis for the hepatitis B vaccination strategy for this specific population.

## Materials and methods

### Population studied

**Figure 1:**
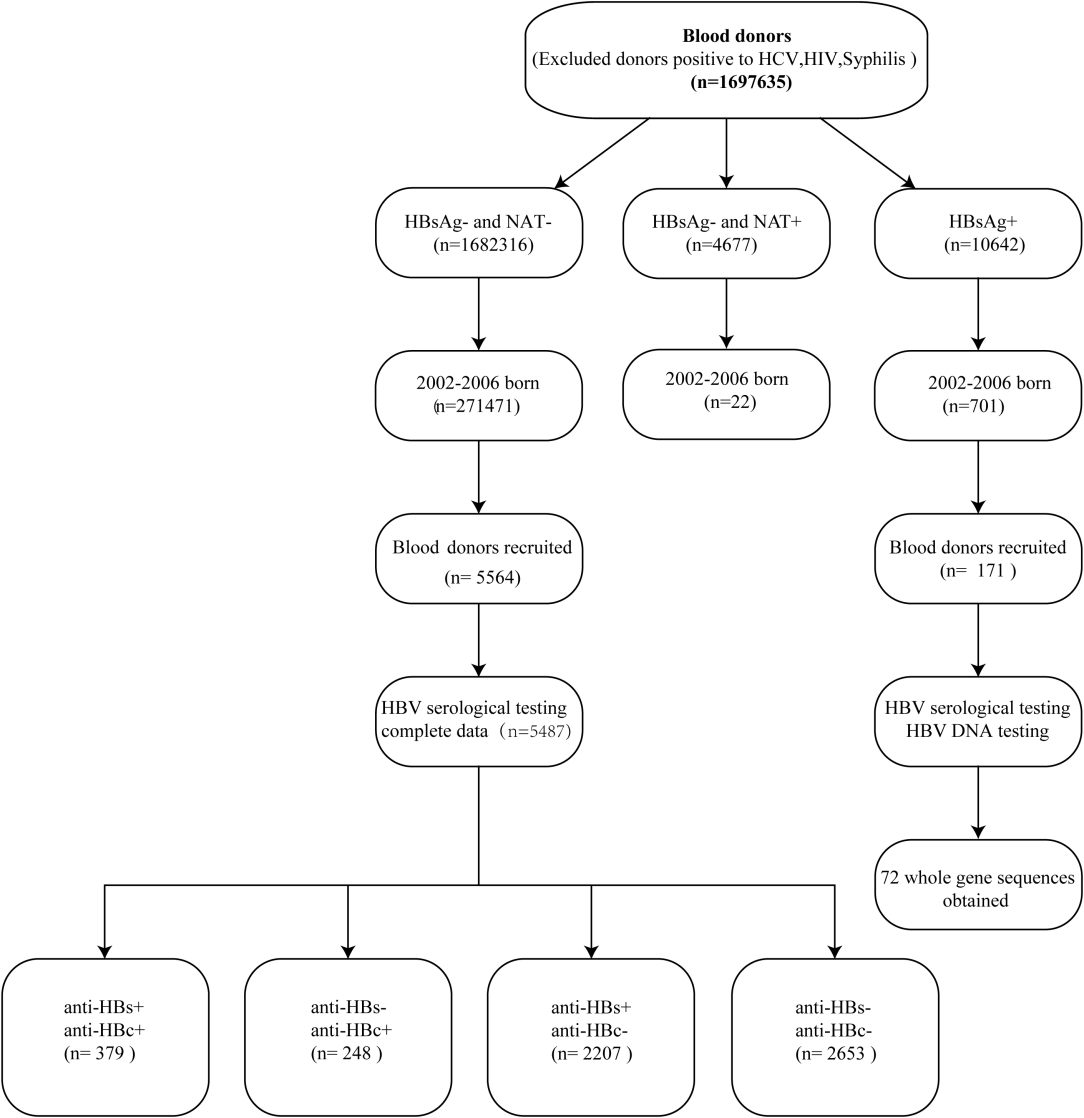
Detection and classification of qualified blood donors and HBV infection in HBsAg+ blood donors. HBV serological testing was performed by electrochemiluminescence immunoassay (ECLIA) for HBsAg, anti-HBs, anti-HBc, HBeAg, and anti-HBe. In addition, HBV DNA testing was performed by real-time quantitative polymerase chain reaction (qPCR) and PCR for long fragment.

This cross-sectional study involved blood donors born in Guangdong between 2002 and 2006 who completed the full course of hepatitis B vaccination. Vaccination status was confirmed either by reviewing the vaccination booklet or through telephone follow-ups. Blood donation in China follows the national Blood Donation Law and technical guidelines. Qualified blood donors are generally healthy adults aged 18-55 (or up to 60 for previous regular donors), with a body weight ≥ 50 kg for males and ≥ 45 kg for females. They must pass a pre-donation health screening (including medical history inquiry and basic physical examination) and meet hemoglobin, blood pressure, and other health standards. The absence of HBsAg and antibodies against infections caused by human immunodeficiency virus (HIV), hepatitis C virus (HCV), and Treponema pallidum (TP) was confirmed, accompanied by normal alanine aminotransferase (ALT) levels. HBsAg and antibodies to HIV, HCV, and TP were tested by individual donation enzyme immunoassays (EIAs). The qualified blood donors were further screened for HBV, HCV, and HIV genomes by nucleic acid testing (NAT) using the Procleix Ultrio Plus Multiplex Assay, followed by screening with the HBV Discriminatory Assay (Grifols Diagnostic Solutions, Inc.) on the Tigris platform. The lower detection limits of these two NAT assays were 3.4 IU/mL and 4.1 IU/mL, respectively. The study population comprised two cohorts recruited from the Guangzhou Blood Center between January 2020 and September 2025: 5,564 qualified donors negative for HBsAg and NAT, and 171 available HBsAg-positive donor samples included in the final analysis (**Figure 1**). All participants were duly informed about this study, and written informed consent was obtained from each participant. All procedures performed in this study involving human participants were in accordance with the 1964 Helsinki declaration and its later amendments or comparable ethical standards. This study was approved by the Medical Ethics Committee of Guangzhou Blood Center.

### HBV serological testing

HBV serologic markers (HBsAg, anti-HBs, HBeAg, anti-HBe, and anti-HBc) were analyzed by a highly sensitive electrochemiluminescence immunoassay [(ECLIA), cobas e602; Roche Diagnostics, Mannheim, Germany] according to the manufacturer’s instructions. HBsAg < 0.05 IU/mL and anti-HBs < 10 IU/L, HBeAg S/CO < 1, or anti-HBe and anti-HBc S/CO > 1 were considered negative.

### HBV DNA quantification, amplification, sequencing, and phylogenetic analysis

The HBV DNA was extracted from 2.5 mL of plasma, using a large volume of high-purity virus nucleic acid extraction kit (Roche Diagnostics, Germany) (9, 20). Viral load was quantified by qPCR with a sensitivity of 2 IU/mL. Viral DNA was also amplified by nested PCRs targeting two HBV long fragments [the fragment A, nucleotide (nt) 3194 to 1797 and the fragment B, nucleotide (nt) 1777-274] as described previously (9, 20–22). Samples positive with qPCR or nested PCR tests were considered HBV DNA positive. Samples negative with qPCR and nested PCR tests were considered HBV DNA not confirmed. The HBV long PCR fragments products were ligated into a cloning vector, which was used for the transformation of *E. coli*, followed by cultivation overnight and five clones were picked up for sequencing. We obtained the full-length genome sequence by combining the two overlapping fragments. MAFFT version 7 (https://mafft.cbrc.jp/alignment/software/) was used to generate the multiple sequence alignment. Phylogenetic trees were constructed by the maximum-likelihood (ML) method in MEGA XI. The ML corrected distances (using the GTR+G+I substitution model) were determined with 1000 iterations of bootstrap sampling. HBV subgenotype reference sequences (23) were downloaded from the National Center for Biotechnology Information (NCBI) database (https://www.ncbi.nlm.nih.gov/nuccore/). HBV genotypes and subgenotypes were confirmed by the phylogenetic tree.

### HBV sequence analyses

A consensus sequence was obtained from each HBV plasma. Consensus sequences were used for alignment analyses (20). BioEdit software (https://bioedit.software.informer.com/) was used to calculate the intragroup variability based on “Sequence Difference Count Matrix” function. HBV wild-type sequences (58 gtB and 23 gtC) were used as controls, which were obtained from HBsAg+ strains selected from blood donors born before 2002 in Guangzhou Blood Center. The GenBank accession numbers are OM669567-OM669647. Average intragroup variability was calculated as the number of nucleotides (regulatory region sequences) or amino acid (protein sequences) substitution differences between study HBV strain sequences and control HBV strain sequences of different genotypes. Point mutations were investigated between study group and control group throughout the four open reading frames (ORFs, Pre-S/S, Pol, Pre-Core/Core, and X region). The HBV-related point mutations in study sample sequences that were not present in any of the reference isolates were designated as uncommon mutations (24). GenBank accession numbers of the full-length HBV genomic sequences from 72 HBsAg+ samples in this study were PX853735-PX853796.

### Statistical analysis

All statistical analyses were performed using SPSS 26.0 software (SPSS, Chicago, IL, USA) and SAS 9.4 software (SAS Institute Inc. Cary, NC, USA). Intergroup variability analyses were performed using the non-parametric Mann-Whitney U test. T-test was used to compare two groups of continuous variables, and the non-parametric Mann-Whitney U test was used when the condition of t-test was not satisfied. The significance of differences in point mutations between two groups was determined using the Fisher’s exact test. Two groups of classifying variables were compared using the χ2 test or Cochran-Mantel-Haenszel tests. The trend analysis of classification variables was performed by Cochran-Armitage trend test. A P-value of < 0.05 was considered statistically significant. Data analysis was performed using GraphPad Prism 10 software.

## Results

### The general characteristics of the qualified blood donors

A total of 5,487 qualified blood donors with complete anti-HBs and anti-HBc data were included in this study (77 individuals with incomplete data were excluded). The selected study population consisted of 3392 males and 2095 females, with an average age of 21.2 years. Among them, there were 2,775 (50.57%) repeat donors and 2,712 (49.43%) first-time donors. Overall, the prevalence of anti-HBs and anti-HBc was 47.73% and 10.58% in males, 44.77% and 12.79% in females, respectively. From 2002 to 2006, the prevalence of anti-HBs in donors was 54.55%, 49.41%, 45.17%, 44.42% and 40.23% respectively, while the prevalence of anti-HBc in donors was 11.39%, 11.53%, 11.40%, 11.49%, and 11.13%. A significant difference in prevalence between males and females was observed in the overall population. The prevalence of anti-HBc was higher in females than in males, whereas the prevalence of anti-HBs showed a positive correlation with age (P < 0.05, **Table 1**). Following the exclusion of anti-HBc+ participants, the trend of rising anti-HBs seroprevalence with advancing age was reaffirmed (P < 0.0001, **Supplementary Table 2**). Moreover, disparities in anti -HBs across distinct regions were also identified. The prevalence of anti-HBs in the Pearl River Delta region (56.54%) was significantly higher than that in the eastern (39.51%), western (44.23%) and northern (43.40%) regions of Guangdong Province (P < 0.001, **Table 2**). The results show that the birth cohort composition is similar between the regions, with no statistically significant differences (P > 0.05, **Supplementary Table 1**).

**Table 1:** Demographic data and HBV serological markers on the population.

Year of birth	Male			Female			Total		
	N	Anti-HBs (%)	Anti-HBc (%)	N	Anti-HBs (%)	Anti-HBc (%)	N	Anti-HBs (%)	Anti-HBc (%)
2002	574	319 (55.57)	63 (10.98)	295	155 (52.54)	36 (12.20)	869	474 (54.55)	99 (11.39)
2003	873	435 (49.83)	91 (10.42)	558	272 (48.75)	74 (13.26)	1431	707 (49.41)	165 (11.53)
2004	862	384 (44.55)	87 (10.09)	577	266 (46.1)	77 (13.34)	1439	650 (45.17)	164 (11.40)
2005	742	338 (45.56)	78 (10.51)	494	211 (42.71)	64 (12.96)	1236	549 (44.42)	142 (11.49)
2006	341	143 (41.94)	40 (11.73)	171	63 (36.84)	17 (9.94)	512	206 (40.23)	57 (11.13)
Total	3392	1619 (47.73)	359 (10.58)	2095	967 (46.16)	268 (12.79)	5,487	2586 (47.13)	627 (11.42)

The differences in the prevalence of anti-HBs and anti-HBc between males and females were determined by Cochran-Mantel-Haenszel test.

Comparison of anti-HBs prevalence between the male and female donors (χ2 = 1.285, p =0.266).

Comparison of anti-HBc prevalence between the male and female donors (χ 2 = 6.242, p =0.013).

The trend analysis in the prevalence of anti-HBs and anti-HBc in different year of birth groups were determined by Cochran-Armitage trend test, respectively. χ2 = -6.732, p <0.0001; χ2 = -0.418, p=0.676.

**Table 2:** HBV serological markers in different regions.

Regions	N	Anti-HBs(%)	χ 2	P	Anti-HBc(%)	χ 2	P
Pearl River Delta	1857	1050 (56.54)*	100.14	<0.0001	226 (12.17)	1.57	0.67
Eastern Guangdong	1263	499 (39.51)			138 (10.93)		
Western Guangdong	1178	521 (44.23)			131 (11.12)		
Northern Guangdong	1189	516 (43.40)			132 (11.10%)		

Comparison between groups was performed by chi-square(χ2). Statistical analysis revealed significant differences in anti-HBs positivity rates among the four regions. Following Bonferroni correction, pairwise comparisons indicated that the positivity rate in the Pearl River Delta region was significantly higher than in the other three regions.

### Characteristics of anti-HBs levels in donors

**Figure 2:**
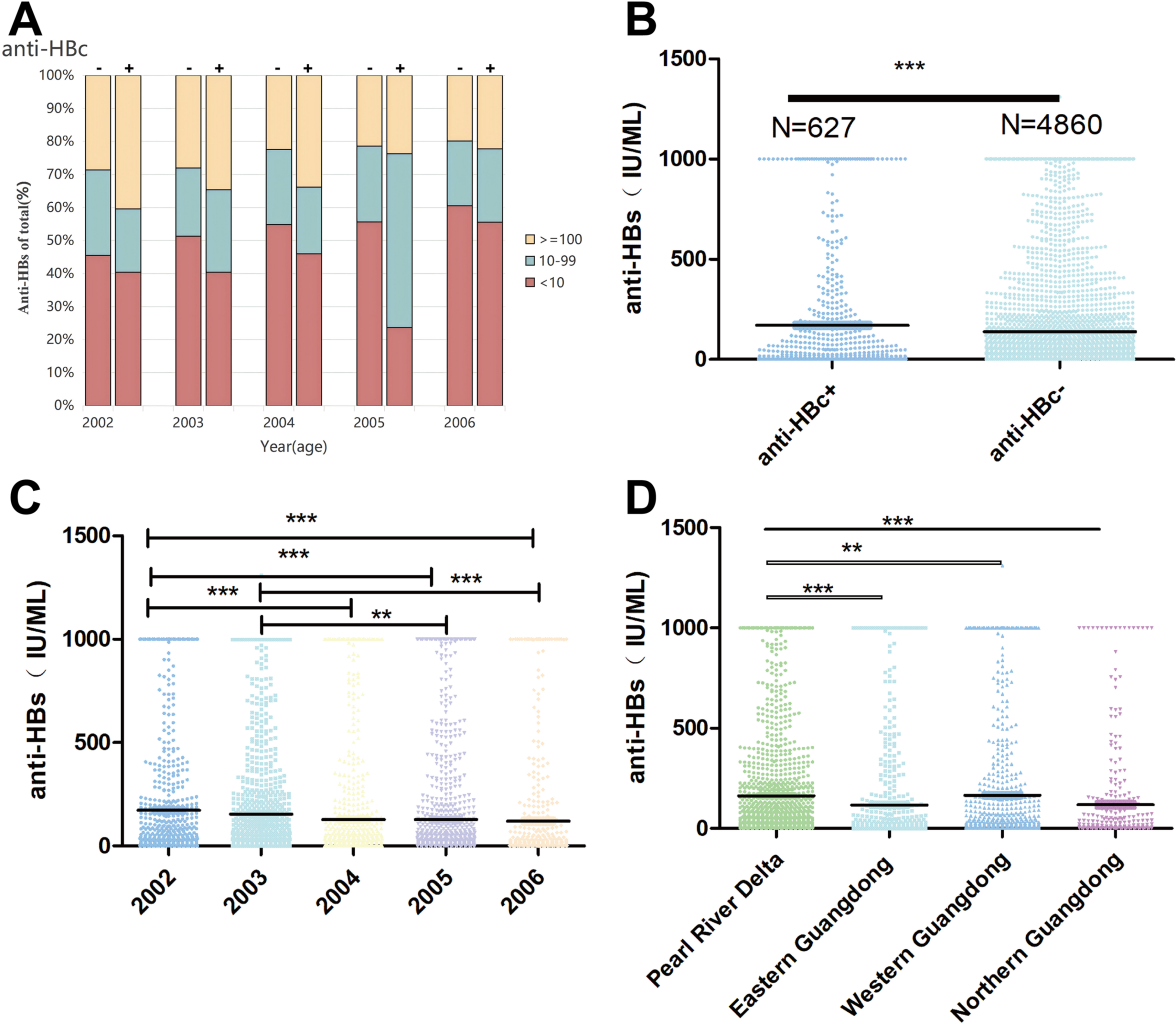
Distribution of anti-HBs level in different group. **(A)** Distribution of anti-HBs reactivity in 627 anti-HBc+ and 4860 anti-HBc- young repeat blood donors according to age. **(B)** Anti-HBs level distribution in anti-HBc+ and anti-HBc- group **(C)** Anti-HBs level distribution between 2002–2006 year of birth **(D)** Anti-HBs level distribution in different regions of Guangdong. Non-parametric Mann-Whitney U test was used for two-group comparison. *indicates P<0.05, ** indicates P<0.01, and *** indicates P<0.001.

The 5,487 samples were divided into two groups based on anti-HBc status: negative and positive (**Figure 2A**). The distribution of anti-HBs levels in the anti-HBc positive group was as follows: 39.55% (248/627) had undetectable anti-HBs levels (< 10 IU/L), 28.71% (180/627) had a low level (10–99 IU/L), and 31.74% (199/627) had a high level (≥100 IU/L). In contrast, the distribution of anti-HBs levels in the anti-HBc negative group differed from that in the anti-HBc positive group. Specifically, 53.17% (2584/4860) had undetectable anti-HBs levels (< 10 IU/L), 22.41% (1089/4860) had a low level (10–99 IU/L), and 24.42% (1187/4860) had a high level (≥ 100 IU/L). Moreover, the anti-HBs level in the anti-HBc positive group was significantly higher than that in the anti-HBc negative group (**Figure 2B**, P < 0.0001).

Furthermore, blood donors born in 2002 and 2003 had significantly higher anti-HBs levels than those born between 2004 and 2006 (**Figure 2C**, P < 0.0001). The significant correlation between age and anti-HBs level persisted even after the exclusion of anti-HBc positive individuals. Additionally, the level of anti-HBs among blood donors in the Pearl River Delta region was significantly higher than that in the eastern, western, and northern regions of Guangdong Province (**Figure 2D**, P < 0.0001).

**Figure 3:**
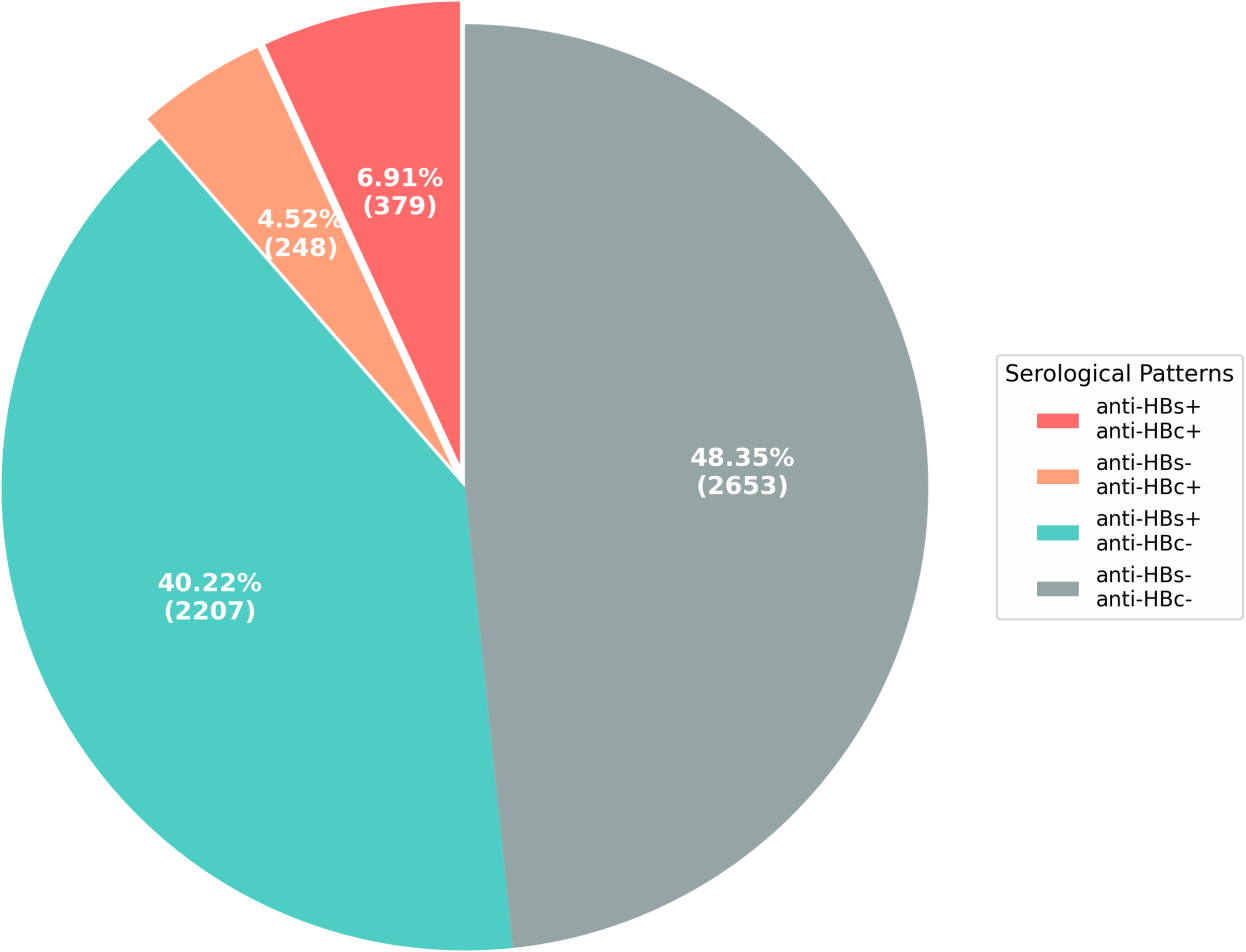
Distribution of HBV serological markers in qualified young blood donor population. Interpretation of Hepatitis B Virus Serological Markers: (1) isolated anti-HBs+ indicates vaccine-induced immunity; (2) anti-HBc+ and anti-HBs+ indicates recovered from past infection (3) anti-HBc- and anti-HBs- indicates susceptible, no prior infection. (4) isolated anti-HBc+ suggests: ① Past resolved infection with waning anti-HBs. ② Occult hepatitis B infection. ③ HBsAg mutant variants undetectable by current laboratory assays. ④ False-positive anti-HBc reaction.

Of the 5,487 qualified donors, 379 (6.91%) were positive for both anti-HBs and anti-HBc, 248 (4.52%) were positive for anti-HBc only, 2,207 (40.22%) were positive for anti-HBs only, and 2,653 (48.35%) were negative for both markers (**Figure 3**).

### HBsAg+ sample classification and characterization

From January 2020 to September 2025, a total of 1,697,635 blood donors were screened, and 10,642 tested positive for HBsAg. A total of 171 HBsAg positive donor samples from individuals born between 2002 and 2006 were available and included in the final analysis. The selected study population consisted of 83 males and 88 females, with an average age of 21.59 years. Among them, there were 18 (10.53%) repeat donors and 153 (89.47%) first-time donors. According to the manufacturer’s instructions (Alanine aminotransferase Reagent Kit, Shanghai Huashi Asia-Pacific Biopharmaceutical Co., Ltd.), ALT values less than or equal to 50 U/L are defined as normal regardless of sex. Only six individuals had abnormal ALT levels. These individuals’ viral loads ranged from undetectable to 5.54E + 08 IU/mL, with a median of 69.1 IU/mL. All of them reported having received the hepatitis B vaccine at birth, though the exact timing and brand of doses varied.

### HBV genotyping and identification of notable mutations

**Figure 4:**
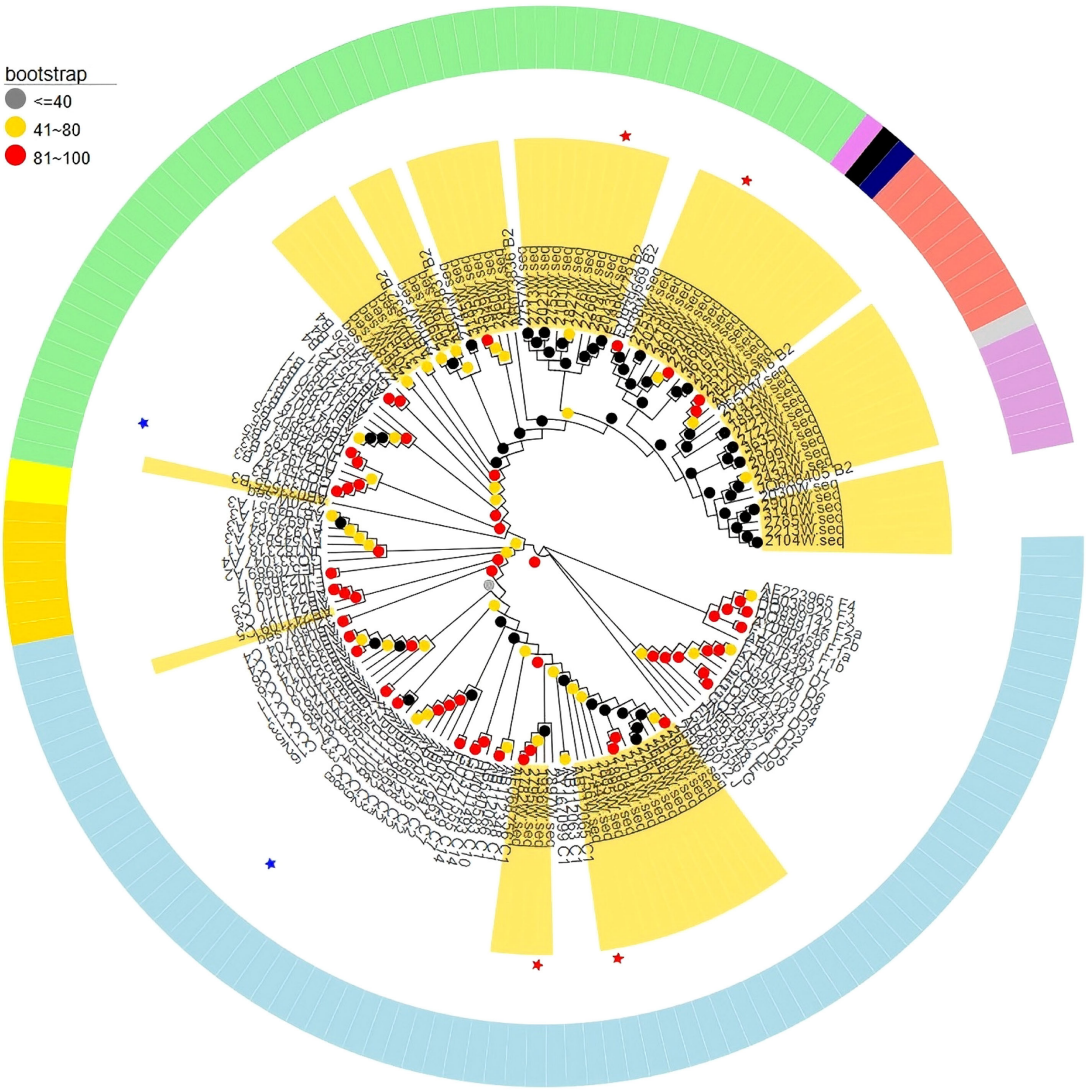
Estimated maximum-likelihood phylogeny for full-length genome sequences of HBV strains. Golden leaves indicate sequences from HBV strains in this study, and the rest indicate reference sequences of proposed genotypes and subgenotypes. Color strips denote different genotypes. Bootstrap analysis values are displayed on the branches. The bars at the middle top of the figure show the scale in nucleotide substitutions per site.

Full-length HBV genome sequences were successfully obtained from 72/171, and genotyping analysis showed that 54/72 (75%) were genotype B, and 18/72 (25%) were genotype C (**Figure 4**). To identify HBV-related point mutations, the amino acid sequences of the genotype B and C strains (excluding strains with deletions and insertions) were compared with their respective controls. Nine HBV-related point mutations were identified in the S, Core, X and Pol genes, as their frequency in individuals positive for HBsAg (born between 2002 and 2006) was significantly higher than in the controls (P < 0.05; **Table 3**). Among them, four mutations have been documented in previous studies (25–29), including L21S, T126A and G145R in the S gene of genotype B, as well as I126T in the S gene of genotype C. The other five mutations were novel findings in genotype B, including G63V in the Core gene, A40T and V44I in the X gene, and G294R and N594H in the Pol gene (**Table 3**).

**Table 3:** Mutations identified in HBV genotype B and C sequences from blood donors.

Region	Mutation	Frequency in HBsAg+ (born in 2002-2006) blood donors (%)	Frequency in HBsAg+ (born before 2002) blood donors (%)	*p*-Value influence
Gt-B				
S	L21S	17/54 (31.48)	8/58 (13.79)	<0.05
	T126A	9/54 (16.67)	2/58 (3.45)	<0.05
	G145R	6/54 (11.11)	0/58 (0)	<0.05
Core	G63V	5/54 (9.26)	0/58 (0)	<0.05
X	A40T	5/50 (10)	0/58 (0)	<0.05
	**V44I**	7/50 (14)	0/58 (0)	**<0.01**
Pol	G294R	4/53 (7.55)	0/58 (0)	<0.05
	N594H	5/53 (9.43)	0/58 (0)	<0.05
Gt-C				
S	I126T	9/18 (50)	4/23 (17.39)	<0.05

The significance of differences in point mutations between two groups was determined using the Fisher’s exact test. Significant diversities are indicated in bold.

**Figure 5:**
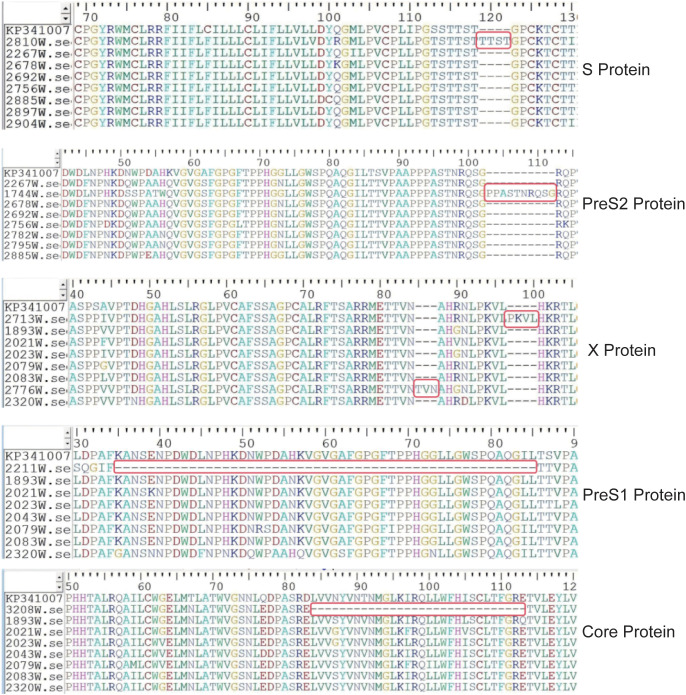
Amino acid location of deletions and insertion in gtB and gtC strains. Deleted or inserted amino acid sequences were labeled with long red boxes. KP341007 was a reference sequence of genotype B.

Insertions and deletions were found in 2 cases and 1 case in genotype B and C strain, respectively (**Figure 5**). Sample 2211 showed a deletion of amino acids 35–85 in the PreS1 gene. Sample 3208 had amino acids 84–113 deleted in the Core gene. Sample 2713 and 2776 had 4 and 3 amino acids insertion in the X gene, respectively. Sample 1744 had a 10-amino-acids insertion (after aa102 in the PreS1 gene), which is located within T/B cell epitopes, and is also the main antigenic region for the production of neutralizing antibodies. Sample 2810 had a 4-amino-acids insertion (after aa118 in the S gene), located in the major hydrophilic region (MHR). Five clones of each strain were sequenced and all supported the deletion/insertion. The deletions and insertions identified in this study have not been reported in other studies (30–36).

## Discussion

HBV infection is a significant public health concern worldwide. China has a relatively high prevalence of chronic HBV infection, with Guangdong Province being one of the most affected regions. Hepatitis B vaccine was fully integrated into the NIP in 2002. Since then, vaccination has been provided free to all infants, ensuring equitable access regardless of socioeconomic status. Consequently, the vaccination coverage rate increased from 30% in 1992 to over 90%, accompanied by a significant decline in HBV prevalence in China (5). Nevertheless, the serological status of individuals vaccinated against HBV as infants remains unclear when they reach adulthood.

This is the first study to demonstrate the epidemiological characteristics of HBV among adult college student blood donors who were vaccinated at birth in Guangdong Province. Serological profiling of 5,487 qualified blood donors revealed that isolated anti-HBs had the highest prevalence (40.22%), indicating predominant vaccine-induced immunity and corroborating the effectiveness of prior immunization initiatives (**Figure 3**). The prevalence of isolated anti-HBc was 4.52%, and 6.91% of the study population were positive for both anti-HBs and anti-HBc. Isolated anti-HBc may signify either a decline in anti-HBs levels after a past infection or potential OBI. Although individuals with isolated anti-HBc often meet standard donation criteria, this subgroup poses a residual risk for transfusion-transmitted HBV, particularly to immunocompromised recipients. The dual-positive pattern (6.91%) is indicative of the convalescent phase, characterized by established immunity and minimal infectivity. Given that 48.35% of donors are susceptible to HBV (dual-negative), and a significant proportion exhibit isolated anti-HBc, reliance on HBsAg screening alone is insufficient to eliminate the risk of transfusion transmission. Implementation of high-sensitivity HBV NAT is therefore essential to mitigate residual risk from OBI and enhance blood safety protocols.

The survey revealed an overall anti-HBs prevalence of 47.13% in the study population (**Table 1**). Compared with previous studies (37–43), the prevalence in our cohort was lower than that reported in Sichuan (50.5%), Nanjing (53.6%), Australia (65.4%), Turkey (55.4%), and the United States (54.0%); higher than figures from Hunan (44.75%) and Singapore (43.3%); and comparable to the rate observed in Poland (47.9%). Overall, a clear downward trend in the prevalence of anti-HBs is observed in most countries. These findings demonstrated that less than half of blood donors in Guangzhou have immunity against HBV, which may be due to the gradual decline in vaccine-induced immunity over time. Notably, this research reveals a higher prevalence of anti-HBs in older college students, likely because more individuals in this group received booster vaccinations or had natural exposure to HBV. Additionally, the median level of anti-HBs (**Figure 2D**) and the prevalence of anti-HBs in the Pearl River Delta region (56.54%) are significantly higher than those in the eastern (39.51%), western (44.23%), and northern (43.40%) regions (**Table 2**). These regional differences may be related to variations in vaccination policies, healthcare access, population density, and other factors (44). The higher prevalence in the Pearl River Delta region is likely due to its more developed economy and better healthcare infrastructure, leading to higher vaccination coverage and timelier booster vaccinations (45).

The prevalence of anti-HBc in infants born between 2002 and 2005 was reported as 3.3%-4.5% (5). This study observed a higher prevalence of 11.42%, indicating that the risk of HBV exposure increased with age. In contrast, the prevalence of anti-HBc in this study population (11.42%) is significantly lower than the 20.9% reported among young blood donors during the early phase of the vaccination program initiated in 1992 (9), likely reflecting a reduced exposure risk due to improved universal vaccination coverage. These findings suggest that universal hepatitis B vaccination during the neonatal period effectively reduces the risk of HBV infection. Nevertheless, whether this protective effect persists into adulthood warrants re-evaluation. Consistent with China’s HBV vaccination policy, booster vaccination is recommended for this population to reduce the risk of infection. Notably, higher among anti-HBc positive donors than among anti-HBc negative donors. The interpretation of anti-HBs levels should therefore consider anti-HBc status: anti-HBc positivity indicates past exposure to HBV, whereas anti-HBs in the absence of anti-HBc typically signifies vaccine-induced immunity. This suggests that anti-HBc positive donors had been exposed to HBV before blood donation, which triggered a secondary immune response, leading to elevated anti-HBs levels (**Figure 2A**). Anti-HBc seroconversion is considered an important serological marker of recovered or active HBV infection, regardless of vaccination status, or OBI (38, 46, 47). The presence of OBI, even at low levels, can pose risks to blood transfusion safety (48). Therefore, the implementation of NAT remains essential to ensure the safety of the blood supply.

HBV is prone to mutations in multiple genomic regions. This study identified nine point mutations significantly associated with immune breakthrough positive individuals in the HBV genome, distributed across the S, Core, X, and Pol gene regions. Among these, four mutations (L21S, T126A, G145R in S gene type B, and I126T in S gene type C) have been previously reported. Notably, G145R, T126A, and I126T mutations are among the most frequent “vaccine escape mutations,” located in the major hydrophilic region (MHR) of HBsAg. These mutations reduce HBsAg synthesis and viral particle secretion, significantly impair neutralizing antibody responses and potentially compromise vaccine efficacy (49–56). These mutations occur more frequently in type B strains, suggesting a potential transmission advantage in specific populations. Additionally, five newly identified mutations (G63V in the Core gene, A40T and V44I in the X gene, G294R and N594H in the Pol gene) exhibit significantly elevated frequencies in type B strains, indicating their possible association with enhanced viral adaptability or immune stress. The G63V mutation within the Core protein is situated in the immunodominant epitope region of hepatitis B core antigen (HBcAg). This mutation may impede the recognition of cytotoxic T lymphocytes (CTLs) and thus promote immune evasion and persistent viral infection. The A40T and V44I mutations in the X protein, which functions as a transcriptional regulator of HBV, may regulate viral replication efficiency or host cell signaling pathways, warranting further functional studies. The G294R and N594H mutations in the Pol gene are located in the reverse transcription domain. Although these are not classic drug resistance sites, they may influence viral replication fidelity or nucleoside analog binding, and their impact on drug susceptibility requires phenotypic analysis.

This study reports multiple insertion and deletion events for the first time, all verified through cloning sequencing to ensure authenticity. Deletions in the PreS1 region (aa 35–85 deletion in sample 2211) may impair viral binding affinity to hepatocyte surface receptors, thereby regulating viral entry. Deletions in the Core region (aa 84–113 deletion in sample 3208), located at the C-terminal of HBcAg, are involved in nucleocapsid assembly and RNA packaging; such deletions may reduce viral particle stability or compromise replication capacity (57). Insertion events in the X region (insertions of 3–4 amino acids in samples 2713 and 2776) may affect the transactivation function of the X protein and alter its interaction with host proteins. These changes can modulate viral replication and carcinogenic potential (58, 59). Sample 1744 is of particular significance, containing a 10-amino-acid insertion after aa 102 in the PreS1 region. This region is rich in T/B cell epitopes and represents a primary target for neutralizing antibody production. Such insertions may alter antigen conformation, impairing antibody recognition and potentially enabling an “immune escape” mechanism (60, 61). Similarly, sample 2810 features a 4-amino-acid insertion after MHR in the S gene, where structural changes could directly impact diagnostic kit sensitivity and vaccine-induced antibody neutralization. Given the lack of reported evidence for such insertions/deletions, their functions need further validation using *in vitro* infection models, pseudovirus neutralization assays, and clinical follow-up data.

This study has several limitations. First, as the participants were recruited solely from Guangzhou, future multi-center studies across diverse regions of China are needed to validate these results. Second, the absence of comprehensive vaccination histories-including precise booster timing and maternal infection status-prevented a detailed assessment of protective durability. Third, our analysis of immune-escape mutations was based on a small sample size; large-scale sequencing studies are required to robustly assess the prevalence and clinical impact of these variants on vaccine efficacy. Additionally, potential risk factors for OBI among young donors were not thoroughly investigated. Despite these limitations, our findings provide valuable preliminary data on the serological status of HBV among young adult blood donors in Guangzhou and highlights the need for continued investigation in this area.

In conclusion, this study provides insights into HBV genetic diversity, expanding the known spectrum of viral variation and revealing the adaptive potential of novel mutations, with potential impacts on transmission dynamics, vaccine effectiveness, and clinical outcomes. Young adults vaccinated in infancy warrant particular attention, as they may exhibit waning immunity. Given the increased risk of exposure during college years, unprotected individuals are at risk of acquiring infection, subsequently contributing to MTCT. These findings support sustained surveillance and targeted booster vaccination strategies for this demographic, which offer significant public health value in preserving population immunity and interrupting transmission cycles (62, 63).

## Data Availability

The datasets presented in this study can be found in online repositories. The names of the repository/repositories and accession number(s) can be found in the article/**Supplementary Material**.
