# Supplementary material for: Hepatitis B vaccination in Chinese infants (2002-2006, Guangdong): long-term effectiveness in adulthood and biological features of HBV infection
Source: Front Immunol. 2026 Apr 29;17:1787290. doi: 10.3389/fimmu.2026.1787290 (PMC13167707; doi:10.3389/fimmu.2026.1787290)
Supplement: Supplementary file 1 [file DataSheet1.pdf]

Table 1. Distribution of birth cohorts from different regions.

**Regions**	2002	2003	2004	2005	2006	Total	**x2**	P
Pearl River Delta（%）	301（16.20）	480（25.83）	469（25.26）	430（23.14）	177（9.53）	1857	18.82	0.09
Eastern Guangdong（%）	214（16.81）	331（26.00）	319（25.06）	283（22.23）	126（9.90）	1273		
Western Guangdong（%）	195（16.70）	323（27.65）	310（26.54）	238（20.38）	102（8.73）	1168		
Northern Guangdong（%）	184（15.48）	287（24.16）	316（26.58）	285（23.99）	117（9.85）	1189		

Note: Comparison between groups was performed by chi-square(χ2).The results show that the age structure and birth cohort composition are similar between the regions, with no statistically significant differences(P>0.05).

Table 2 . Demographic data and HBV serological markers on the population.

Year of birth	Male			Female			Total	
	N	anti-HBs+ anti-HBc-(%)	anti-HBs- anti-HBc-(%)	N	anti-HBs+ anti-HBc-(%)	anti-HBs- anti-HBc-(%)	N	anti-HBs+(%)
2002	511	296(57.93)	215(42.07)	259	135(52.12)	124(47.88)	770	431(55.97)
2003	799	395(49.44)	404(50.56)	476	219(46.01)	257(53.99)	1275	614(48.16)
2004	795	352(44.28)	443(55.72)	505	223(44.16)	282(55.84)	1300	575(44.23)
2005	670	301(44.93)	369(50.07)	430	182(42.33)	248(57.67)	1100	483(43.91)
2006	295	124(42.03)	171(57.97)	154	64(41.56)	90(58.44)	449	188(41.87)
Total	3070	1468(47.82)	1602(52.18)	1824	823(45.12)	1001(54.88)	4894	2291(46.81)

Note:The differences in the prevalence of anti-HBs between males and females were determined by Cochran-Mantel-Haenszel test.

Comparison of anti-HBs prevalence between the male and female donors(χ 2 = 2.75, p = 0.10).

The trend analysis in the prevalence of anti-HBs different year of birth groups were determined by Cochran-Armitage trend test.(χ 2 = -6.924, p<0.0001)
